# Efficacy of *Lactococcus lactis* WiKim0124 in Fat-, Sucrose-, and Fat/Sucrose-Induced Obesity Models

**DOI:** 10.1038/s41538-026-00915-3

**Published:** 2026-06-04

**Authors:** Moeun Lee, Daun Kim, Jung Hee Song, Shin Jae Park, Ji Yoon Chang

**Affiliations:** 1 Sustainable Distribution Research Group, World Institute of Kimchi, Gwangju, 61755 Korea; 2 Intelligent Fermentation Research Group, World Institute of Kimchi, Gwangju, 61755 Korea; 3 Department of Food and Nutrition, Chosun University, Gwangju, 61452 Republic of Korea; 4 ShinWoo Co., Ltd. (Headquarters), 991 Warasan-ro, Munsan-eup, Jinju-si, Gyeongsangnam-do 52839 Republic of Korea; 5 Division of Applied Life Science (BK21), Institute of Agriculture and Life Science, Gyeongsang National University, Jinju, 52828 Republic of Korea; 6 Institute of Smart Space Agriculture (ISSA), Gyeongsang National University, Jinju, Gyeongnam 52828 South Korea

**Keywords:** Diseases, Gastroenterology, Microbiology

## Abstract

*Lactococcus lactis* WiKim0124 (WiKim0124), a probiotic strain isolated from kimchi, has previously shown anti-obesity effects in high-fat diet (HFD) models. This study investigated whether WiKim0124 and its formulated version, SW01, exert consistent anti-obesity efficacy across distinct diet-induced obesity models through modulation of host lipid metabolism and gut microbial function. In 3T3-L1 adipocytes and FFA-treated HepG2 cells, both treatments inhibited lipid accumulation and modulated lipid metabolism–related markers, indicating enhanced fatty acid oxidation and reduced lipogenesis. In C57BL/6 J mice fed HFD, high-sucrose (HSuc), or HFD + HSuc diets, daily oral administration of WiKim0124 or SW01 significantly reduced body weight gain, adipose tissue mass, and hepatic lipid accumulation. WiKim0124 and SW01 significantly enhanced fatty acid oxidation pathways, as evidenced by increased expression of the markers PPARα, CPT-1α, and UCP2. Gut microbiota analysis showed increased Bacteroidetes and enrichment of *Akkermansia muciniphila* in treated groups. Shotgun metagenomic functional profiling revealed enhanced short-chain fatty acid–related pathways and enzymes, with distinct patterns depending on treatment and dietary stressors. Microbial functional responses were most pronounced in the HFD + HSuc model, supporting a diet-dependent mode of probiotic action. Together, these findings demonstrate consistent anti-obesity efficacy of WiKim0124 and support the translational potential of its formulated application through integrated modulation of host metabolism and gut microbial function.

## Introduction

Obesity has emerged as a leading global health concern in the 21st century, with its prevalence nearly tripling since 1975^1^. Based on World Health Organization data from 2016, 39% of adults worldwide were categorized as overweight, while 13% met the criteria for obesity^2^. It is closely linked to numerous health complications, such as insulin resistance, metabolic syndrome, and non-alcoholic fatty liver disease^3^. While genetic and epigenetic factors contribute to its development, other elements—including lifestyle habits, appetite regulation, hormonal signaling, and immune system function—also play significant roles^2^. Among these, an imbalance between caloric intake and energy expenditure, particularly due to high-fat and high-sugar diets, has been identified as a principal driver of excessive weight gain^4^.

The gut microbiota plays a pivotal role in maintaining host health by directly influencing the digestion, absorption, and metabolism of nutrients^5^. Certain probiotic strains, for instance, ferment indigestible dietary carbohydrates to produce microbial metabolites such as short-chain fatty acids (SCFAs), which contribute to host physiological regulation and immune homeostasis. These SCFAs have been associated with protective effects against inflammatory and metabolic disorders^6^. The composition of the gut microbiota is influenced by multiple factors, including dietary habits and lifestyle choices, and accumulating evidence indicates that individuals with obesity tend to exhibit reduced microbial diversity and altered community structures compared to healthy individuals^7^.

Kimchi, a staple fermented food in Korean cuisine, is made by fermenting vegetables with a blend of spices^8^. Numerous studies have explored the health-promoting effects of its bioactive components, particularly focusing on kimchi-derived lactic acid bacteria (LAB), which have demonstrated immunoregulatory, anti-obesity, antidiabetic, and anticancer properties^9^. Furthermore, exopolysaccharides produced by these LAB strains have been shown to possess significant antioxidant activity^10^. Recently, probiotics isolated from kimchi—including both live and heat-killed strains, as well as their components and metabolites—have attracted growing attention as novel strategies for preventing and mitigating obesity and related chronic diseases^11^. This interest is largely driven by their potential to influence lipid, glucose, and cholesterol metabolism, modulate the intestinal microbiota, and exert antioxidant and anti-inflammatory effects^12^. In our previous study, we demonstrated that *Lactococcus lactis* WiKim0124, a strain isolated from kimchi, exerted anti-obesity and anti-inflammatory effects in mice with high-fat diet-induced obesity^13,14^. However, the mechanisms by which this strain modulates gut microbiota composition, and how these changes relate to obesity and its associated metabolic disorders, remain unclear.

Therefore, this study aimed to (i) investigate how WiKim0124 modulates the gut microbial structure in mice fed a high-fat/high-sucrose diet, (ii) explore the relationship between gut microbiota alterations and obesity-related phenotypes, and (iii) evaluate whether anti-obesity efficacy is retained following formulation of WiKim0124 as SW01. Additionally, we assessed the degree of weight gain induced by the high-fat/high-sucrose diet and evaluated the efficacy and underlying mechanisms of WiKim0124 in mitigating these effects, thereby providing a theoretical foundation for the development of functional probiotics with anti-obesity potential.

## Results

### Effects of WiKim0124 treatment on differentiated 3T3-L1 and HepG2 cells

**Fig. 1:**
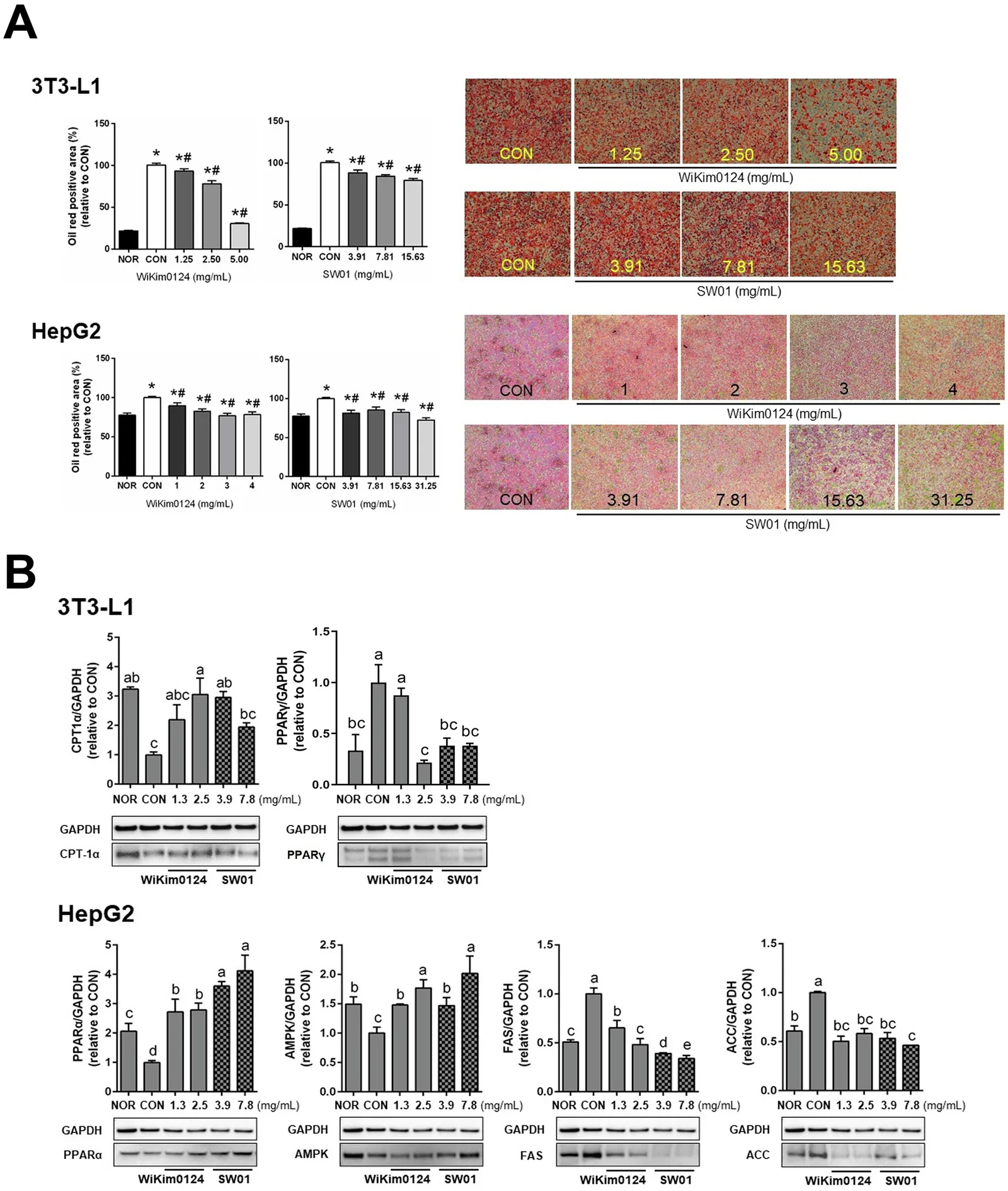
Effects of WiKim0124 and SW01 lysates on 3T3-L1 and HepG2 cells. **A** Quantification of intracellular lipid accumulation by absorbance at 517 nm and representative photomicrographs (×200) following Oil Red O staining. NOR normal control, CON adipogenically differentiated 3T3-L1 cells or free fatty acid-treated HepG2 cells. *p < 0.05 vs. NOR; #*p* < 0.05 vs. CON. Data are expressed as mean ± SD (*n* = 3). **B** Expression of lipid metabolism-related proteins in 3T3-L1 and HepG2 cells. Protein expression levels of CPT-1α, PPARγ, PPARα, AMPK, FAS, and ACC were measured by Western blot analysis. NOR: normal control; CON: adipogenically differentiated 3T3-L1 cells or free fatty acid-treated HepG2 cells. Different letters indicate statistically significant differences between groups (*p* < 0.05, one-way ANOVA followed by Tukey’s HSD test). Data are presented as mean ± SD (*n* = 3).

Treatment with 5 mg/mL LAB lysate and 31.25 mg/mL SW01 samples did not induce any cytotoxicity in either 3T3-L1 or HepG2 cells (Supplementary Fig. S2). To investigate the anti-adipogenic effects of WiKim0124 and its formulated form (SW01), lipid accumulation was assessed in 3T3-L1 and HepG2 cells using the Oil Red O staining assay (Fig. 1A). Treatment with the samples suppressed lipid accumulation in both cell lines, with the WiKim0124 lysate showing the most pronounced effect, followed by SW01. Based on these results, further experiments were conducted at the same concentrations to examine the underlying anti-adipogenic mechanisms and their impact on hepatic fatty acid oxidation pathway in the context of obesity. Western blot analysis was performed on fully differentiated 3T3-L1 adipocytes and free fatty acid (FFA)-treated HepG2 cells to examine the expression of lipid metabolism- and fatty acid oxidation-related proteins. In 3T3-L1 cells, CPT-1α and PPARγ were analyzed. In HepG2 cells, the expression of PPARα, AMPK, FAS, and ACC was evaluated. The expression of each protein was significantly modulated by the treatments. In 3T3-L1 adipocytes, CPT-1α expression was significantly upregulated, while PPARγ expression was significantly downregulated, with the strongest effect observed in the WiKim0124-treated group. In HepG2 cells, the expression levels of PPARα and AMPK were notably increased, while FAS and ACC were downregulated. Similar regulatory effects were observed with SW01, exhibiting a dose-dependent modulatory pattern.

### Impact of WiKim0124-mediated regulation on body weight and fat distribution

**Fig. 2:**
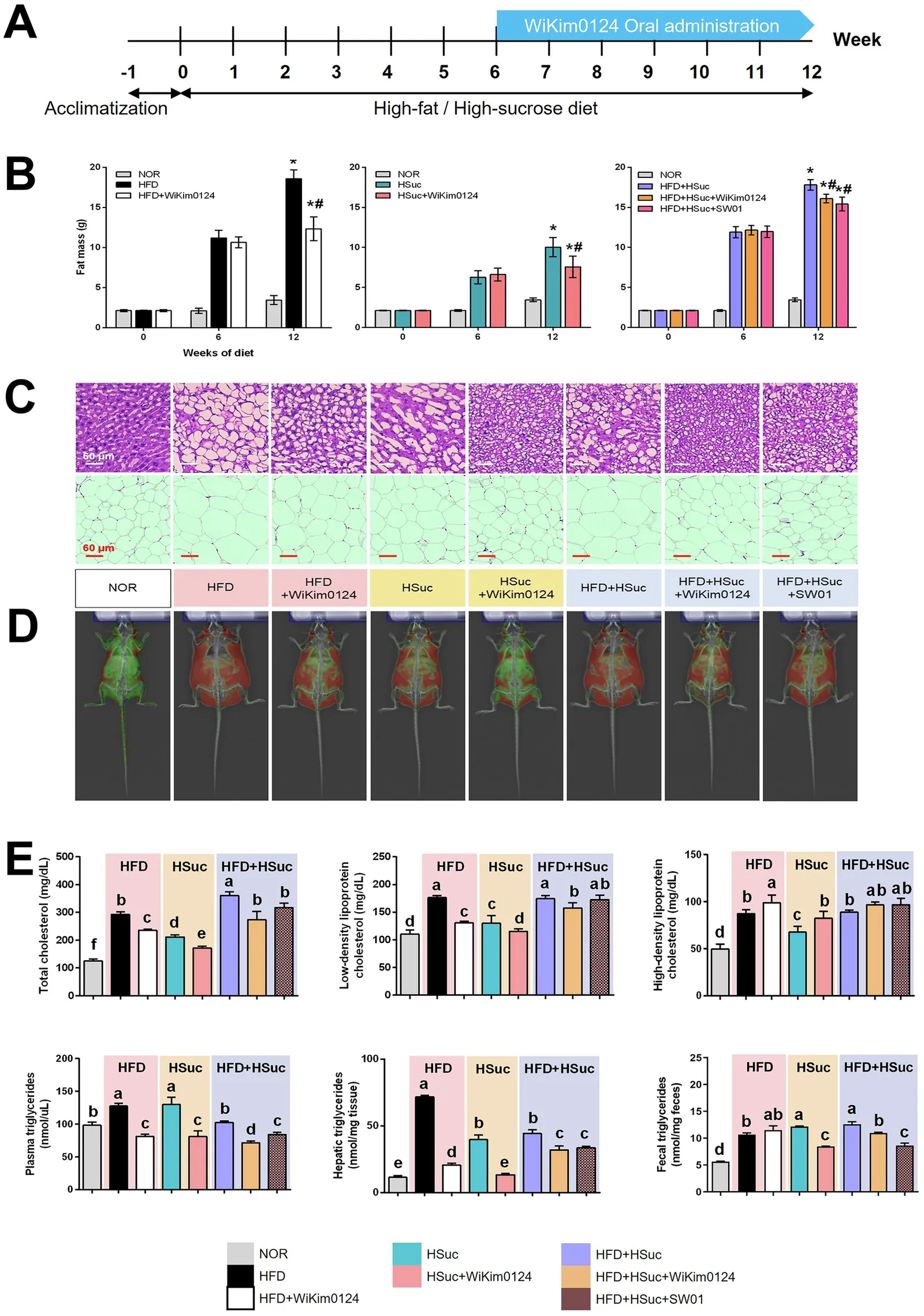
Effects of WiKim0124 and SW01 administration in high-fat diet (HFD)-, high-sucrose diet (HSuc)-, and combined high-fat/high-sucrose diet (HFD+HSuc)-induced obese mice. **A** Schematic overview of the experimental schedule. **B** Fat mass measured at weeks 0, 6, and 12. **C** Representative H&E-stained images of liver and epididymal white adipose tissue sections (20× magnification). **D** Body composition analysis obtained by DXA scan. The DXA images in Fig. 2D are original images generated by the authors. **E** Biochemical measurements: plasma total cholesterol, low-density lipoprotein cholesterol, high-density lipoprotein cholesterol, triglyceride, hepatic triglyceride, and fecal triglyceride. Different letters indicate statistically significant differences among groups (*p* < 0.05, one-way ANOVA followed by Tukey’s HSD test). Values are presented as mean ± SD (*n* = 8).

When mice were fed a diet supplemented with WiKim0124 following obesity induction by HFD, HSuc, or combined HFD + HSuc for 6 weeks, a significant suppression of body weight gain was observed (Supplementary Fig. S1A). Notably, a substantial reduction in fat mass was observed in the WiKim0124- and SW01-treated groups (Fig. 2B), accompanied by a significant increase in lean mass (Supplementary Fig. S1B). While liver, spleen, and kidney weights remained unchanged across groups, significant reductions in epididymal, visceral, and renal fat weights were observed in all obesity-induced mice (Table 1). Notably, in HFD+HSuc-induced mice, both the WiKim0124 and SW01 treatment groups exhibited substantial fat reduction. To assess the impact of WiKim0124 and SW01 on lipid accumulation, liver tissue and epididymal fat were examined following H&E staining (Fig. 2C). In the HFD group, pronounced lipid accumulation was evident in hepatic tissue, accompanied by enlarged adipocytes in epididymal fat. These findings were supported by DXA scan images, which revealed elevated fat content across all obesity-induced groups (Fig. 2D). In contrast, mice treated with either WiKim0124 or SW01 showed a significant reduction in adipocyte size in both liver and epididymal fat tissues, accompanied by decreased overall body fat mass. Furthermore, lipid accumulation was more effectively attenuated in the WiKim0124 group than in the SW01 group. Consistent with these findings, WiKim0124 administration led to reductions in plasma T-CHO and LDL-C across all obesity models. However, no significant differences in lipid-lowering efficacy were observed between the WiKim0124 and SW01 groups. Both treatments elevated HDL-C in the HFD and HSuc groups, but not significantly in the HFD + HSuc model (Fig. 2E). In all obese mouse models, treatment with either sample reduced plasma, hepatic, and fecal TG levels, as well as AST and ALT levels (Supplementary Fig. S1C).

**Table 1:** Organ weights of experimental groups

	NOR	HFD		HSuc		HFD+HSuc			
		PBS	WiKim0124	PBS	WiKim0124		PBS	WiKim0124	SW01
Liver	1.21 ± 0.04	1.37 ± 0.08	1.14 ± 0.04	1.23 ± 0.02	1.12 ± 0.02		1.15 ± 0.02	1.16 ± 0.09	1.10 ± 0.07
Spleen	0.06 ± 0.01	0.07 ± 0.01	0.06 ± 0.00	0.07 ± 0.01	0.06 ± 0.00		0.06 ± 0.00	0.07 ± 0.01	0.07 ± 0.00
Kidney	0.34 ± 0.02	0.31 ± 0.01	0.34 ± 0.01	0.31 ± 0.02	0.31 ± 0.01		0.26 ± 0.01	0.30 ± 0.01	0.30 ± 0.01
Epididymal fat	0.38 ± 0.04	2.65 ± 0.04^*^	2.30 ± 0.10^*#^	1.48 ± 0.11^*^	0.93 ± 0.14^*#^		2.30 ± 0.10^*^	1.70 ± 0.15^*#^	2.11 ± 0.12^*#^
Visceral fat	0.36 ± 0.03	1.18 ± 0.13^*^	0.63 ± 0.05^*#^	0.66 ± 0.05^*^	0.37 ± 0.03^*#^		0.75 ± 0.04^*^	0.59 ± 0.09^*#^	0.65 ± 0.05^*#^
Renal fat	0.06 ± 0.00	0.42 ± 0.09^*^	0.12 ± 0.00^*#^	0.13 ± 0.01^*^	0.08 ± 0.01^*#^		0.25 ± 0.02^*^	0.18 ± 0.01^*#^	0.22 ± 0.01^*#^

*HFD* high-fat diet, *HSuc* high-sucrose diet, *HFD+HSuc* combined high-fat/high-sucrose diet (*p < 0.05 vs. NOR, #*p* < 0.05 vs. PBS).

### Expression of adipogenic-, and fatty acid oxidase-related proteins

**Fig. 3:**
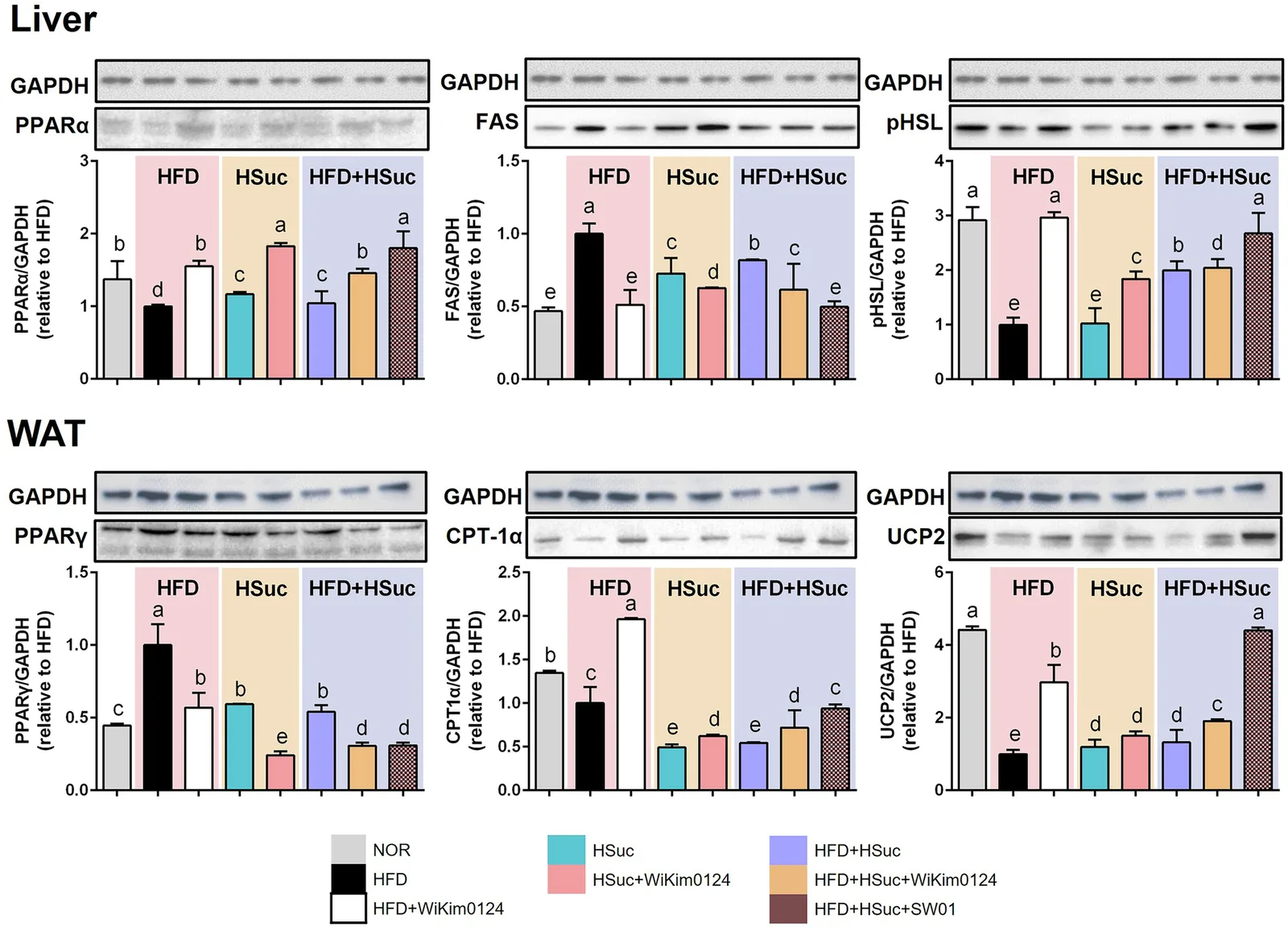
Expression of lipid metabolism–related proteins in mice with diet-induced obesity (HFD, HSuc, and HFD+HSuc models). Protein levels of hepatic PPARα, FAS, and pHSL, as well as white adipose tissue (WAT) PPARγ, CPT-1α, and UCP2, were analyzed by Western blotting. Different letters indicate statistically significant differences among groups (*p* < 0.05, one-way ANOVA followed by Tukey’s HSD test). Values are expressed as mean ± SD (*n* = 3).

To elucidate the underlying mechanisms of the anti-obesity effects of WiKim0124 and SW01 in mice with obesity induced by a HFD, HSuc, or combined HFD + HSuc, the expression of proteins associated with adipogenesis inhibition and fatty acid oxidation in epididymal white adipose tissue and liver tissue was assessed via western blotting (Fig. 3). Both WiKim0124 and SW01 significantly downregulated hepatic expression of FAS and upregulated PPARα levels. In contrast, CPT-1α and UCP2 expression was elevated across all treatment groups, with the degree of upregulation varying depending on the treatment type and dietary condition.

### Impact of WiKim0124 on obesity-related microbial composition

**Fig. 4:**
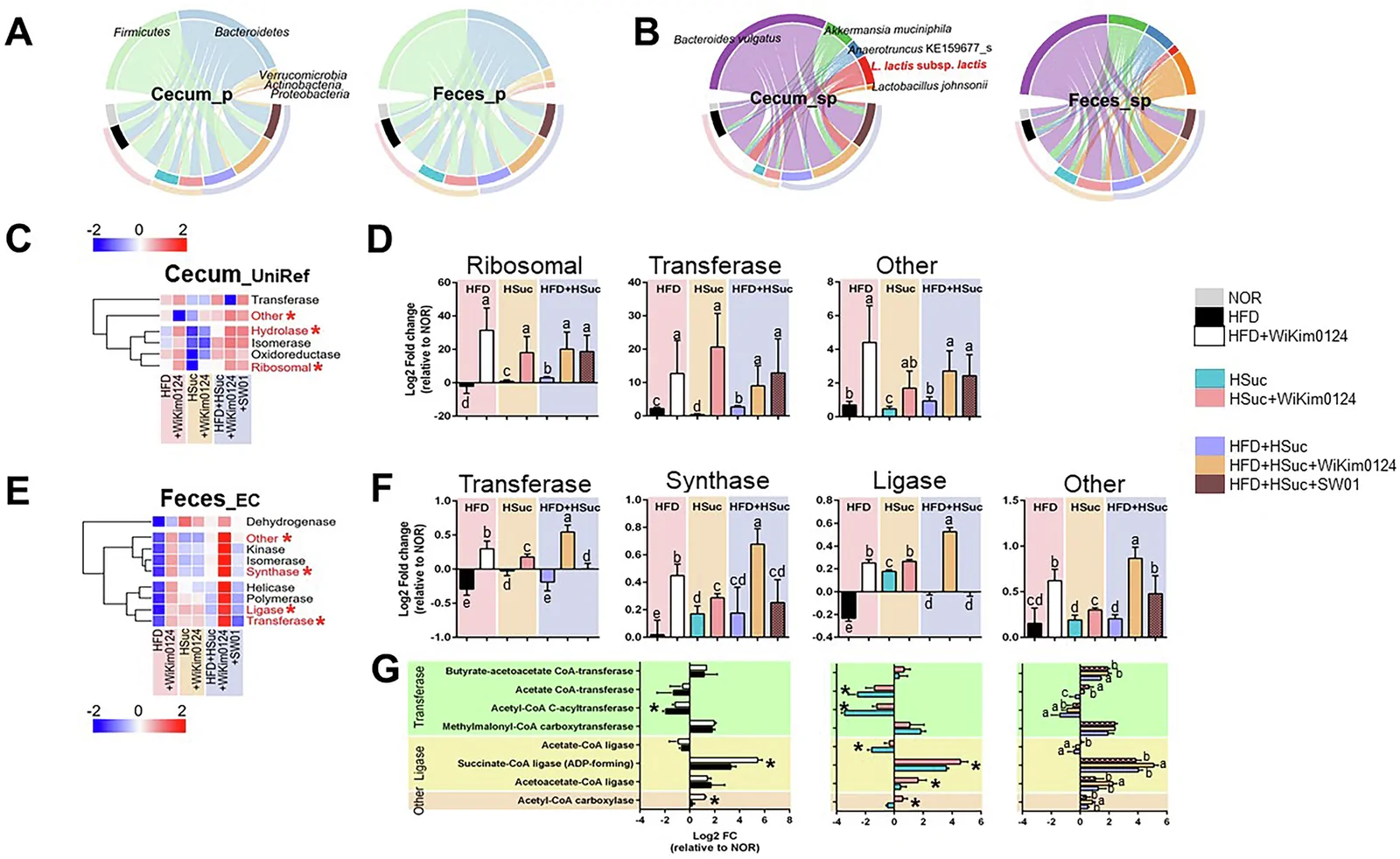
Modulatory effects of WiKim0124 and SW01 on the composition and predicted functions of the gut microbiota in mice with HFD-, HSuc-, and HFD+HSuc-induced obesity. **A** Top 5 microbial phyla in cecal and fecal samples. The outer ring segments represent either experimental groups or the top 5 microbial phyla. The colored chords inside the circle indicate the relative abundance of each phylum within specific experimental groups. **B** Top 5 microbial species in cecal and fecal samples. The outer ring segments represent either experimental groups or the top 5 microbial species, with colored chords denoting their relative abundances per group. **C** Hierarchical clustering of UniRef pathway profiles in cecal samples. Colors represent relative abundances, with red indicating higher intensity and blue indicating lower intensity. **D** Log2 fold change (vs. NOR) in significantly altered UniRef pathways in the cecum across experimental groups. **E** Hierarchical clustering of enzyme commission (EC) profiles in cecal samples. Colors represent relative abundances, with red indicating higher intensity and blue indicating lower intensity. **F** Log2 fold change (vs. NOR) in significantly altered EC categories in fecal samples. **G** SCFA-related EC abundance patterns across groups. Asterisks (*) indicate statistically significant differences (*p < 0.05). Different letters indicate statistically significant differences among groups (*p* < 0.05, one-way ANOVA followed by Tukey’s HSD test). Values are presented as mean ± SD (*n* = 5).

To determine whether the metabolic benefits of WiKim0124 treatment are linked to the restoration of gut microbiota disrupted by HFD, HSuc, or their combination (HFD + HSuc), microbial profiles in cecal and fecal samples were analyzed and compared. In all obesity-induced groups, the Firmicutes-to-Bacteroidetes ratio in both cecal and fecal samples was significantly altered compared to the NOR group. Notably, the relative abundances of *Firmicutes* and *Bacteroidetes* were consistently elevated across all treatment groups, including those receiving WiKim0124 (Fig. 4A). Among the top five dominant species, *Bacteroides vulgatus* and *Akkermansia muciniphila* were markedly increased in all treatment groups compared to the untreated obesity-induced groups, in both cecal and fecal samples. This effect was most pronounced in the HFD + HSuc-induced group, followed by the HFD-induced group. *Lactococcus lactis* subsp. *lactis* was predominantly detected in cecal samples, and its presence was exclusive to the WiKim0124 or SW01 treatment groups. Although *B. vulgatus* and *A. muciniphila* exhibited parallel abundance patterns in fecal and cecal samples, *L. lactis* subsp. *lactis* was present at diminished levels and showed limited detection in fecal microbiota profiles (Fig. 4B).

### Shotgun metagenomic functional profiling in gut microbiota associated with obesity and modulation by WiKim0124

Functional pathway analysis was performed using both UniRef and Enzyme Commission (EC) annotations derived from the cecal and fecal metagenomic datasets. In the UniRef-based analysis, only the cecal dataset showed statistically significant differences between groups (Kruskal–Wallis, *p* < 0.05), with enriched pathways including Ribosomal, Hydrolase, and Other functions (Fig. 4C). In contrast, the EC-based analysis revealed significant differences in the fecal dataset (*p* < 0.05), highlighting functional categories such as Transferase, Ligase, Synthase, and Other (Fig. 4E). Analysis of the log2 fold change in functional categories enriched in the cecal UniRef dataset revealed that pathways such as Ribosomal, Transferase, and Other, which were associated with the NOR group, were significantly increased in all treatment groups compared to the obesity-induced controls. WiKim0124 and SW01 induced similar enrichment patterns in EC-level functional pathways (Fig. 4D). Similarly, in the fecal EC dataset, the functions Transferase, Synthase, Ligase, and Other showed a significant increase in WiKim0124-treated groups relative to the obesity-induced groups. SW01 exerted a selective modulatory effect on Transferase and Other EC functions in the HFD+HSuc model, whereas Synthase and Ligase categories did not show significant changes (Fig. 4F). Among these functional categories, several enzyme annotations associated with short-chain fatty acid (SCFA) metabolism showed significant differences, particularly within the Transferase, Ligase, and Other enzyme groups. Across the HFD+HSuc model, eight SCFA-related enzyme annotations showed significantly increased abundance relative to obesity controls. Among them, annotations corresponding to acetyl-CoA C-acyltransferase and acetate-CoA ligase showed higher abundance in the SW01-treated group, whereas annotations corresponding to succinate-CoA ligase, acetoacetate-CoA ligase, and acetyl-CoA carboxylase showed higher abundance in the WiKim0124-treated group. In the HSuc-induced obesity model, WiKim0124 administration was associated with the second-highest number of significantly enriched SCFA-related enzyme annotations.

## Discussion

In our previous research, WiKim0124 was identified as a promising anti-obesity strain based on its efficacy in both in vitro and in vivo models, including reductions in body weight and modulation of obesity-related gene expression in HFD-induced obese mice^13,15^. Carbohydrates regulate energy intake and contribute to adiposity. Weight gain is primarily influenced by the combined availability of dietary carbohydrates and fats^16^. This study extended the evaluation of WiKim0124 live cells across multiple obesity models induced by HFD, HSuc, and HFD+HSuc, and further assessed a formulated preparation (SW01) containing the same strain to determine response consistency across different delivery formats.

PPARγ is a key transcription factor that regulates adipogenesis and lipid storage, with elevated expression in mature adipocytes^17^. FAS, a key enzyme in de novo lipogenesis, drives fatty acid synthesis and is commonly upregulated in obesity^18^. In contrast, AMPK maintains cellular energy homeostasis by promoting lipid catabolism through phosphorylation. Its activation leads to the phosphorylation of ACC, reducing malonyl-CoA levels—a known inhibitor of CPT1—thereby enhancing mitochondrial fatty acid oxidation^19^. Both WiKim0124 and SW01 effectively reduced lipid accumulation in both 3T3-L1 adipocytes and HepG2 hepatocytes. In 3T3-L1 cells, treatment significantly upregulated CPT-1α and downregulated PPARγ, suggesting enhanced fatty acid oxidation and suppressed adipogenesis. In HepG2 cells, both treatments led to significant upregulation of PPARα and AMPK, along with downregulation of FAS and ACC, indicating a beneficial regulatory effect on hepatic lipid metabolism.

Considering these results, WiKim0124 and SW01 were further evaluated in obese mice models induced by HFD, HSuc, and combined HFD+HSuc. The daily dosage was set at 10^10^ CFU/day for WiKim0124 and 10 mg/kg body weight (equivalent to 10^9^ CFU/day of WiKim0124) for SW01. Both treatments significantly reduced body weight gain and total body fat mass, while also suppressing plasma T-CHO, LDL-C levels, hepatic TG accumulation, and fecal TG content. Dietary TG are a major high-calorie energy source. Pancreatic lipase, the key enzyme for TG digestion, hydrolyzes TGs into absorbable glycerol and fatty acids, which are later re-esterified and stored in tissues, contributing to obesity^20^. Fecal TG levels serve as a marker of intestinal lipid absorption efficiency, which plays a crucial role in obesity. While increased absorption promotes adipose fat accumulation, compounds such as Orlistat mitigate this by enhancing fecal lipid excretion^21^. Our findings suggest that, while lipid absorption appears to be unaffected, WiKim0124 and SW01 may primarily reduce lipid accumulation by enhancing intracellular lipid utilization rather than inhibiting intestinal lipid absorption. Unlike fat absorption inhibitors, these treatments appear to promote fatty acid oxidation, protect the liver from lipid overload, and support systemic lipid homeostasis.

Cholesterol homeostasis, largely regulated by the liver and intestine, is reflected in TC levels^22^. Previous studies have demonstrated the anti-obesity and cholesterol-lowering effects of WiKim0124 in obese mice. In the present study, WiKim0124 and SW01 treatment consistently reduced TC and LDL-C while increasing HDL-C across all obesity models, suggesting a consistent regulatory effect on lipid metabolism. Protein expression analysis revealed upregulation of hepatic PPARα and pHSL and suppression of FAS. pHSL, a key marker of lipolytic activity, reflects the activation of intracellular triglyceride hydrolysis in adipocytes^23^. Meanwhile, PPARα activation drives mitochondrial fatty acid oxidation, contributing to reduced lipid storage^24^. Together, these findings indicate a coordinated shift toward increased lipid breakdown and reduced lipid synthesis, supporting the lipid-lowering effects of the treatment. In parallel within WAT, WiKim0124 and SW01 modulated the expression of key metabolic genes, including the downregulation of PPARγ, and the upregulation of CPT-1α and UCP2, indicating suppressed adipogenesis and enhanced mitochondrial fatty acid oxidation. UCP2 contributes to this shift by dampening glycolysis and facilitating β-oxidation, which helps prevent lipid overload^25^. Although WiKim0124 and SW01 comparably suppressed PPARγ expression, SW01 demonstrated metabolic regulatory effects by upregulating key lipid oxidation markers—PPARα, CPT-1α, UCP2, and pHSL—and downregulating FAS across all obesity models, with efficacy varying by obesity type. These findings suggest that the regulatory effects of WiKim0124 and SW01 on host lipid metabolism may vary depending on the specific dietary stressor used to induce obesity. These results indicate that the modulatory effects of WiKim0124 and SW01 on lipid metabolism are diet-dependent. Notably, microbial functional responses were most pronounced in the HFD + HSuc model, whereas the HFD and HSuc models showed distinct treatment-responsive metabolic features, further supporting a diet-dependent mode of probiotic action. As the role of non-microbial formulation components was not assessed, the study focused on live WiKim0124 dosage, with SW01 used to evaluate efficacy consistency in a formulated format.

Obesity is frequently associated with gut microbiota dysbiosis, particularly a decreased relative abundance of *Bacteroidetes* and *Firmicutes*. This dysbiotic pattern was consistently observed across all obesity models in the present study^26^ and was partially improved following treatment. These observations are broadly consistent with prior studies linking obesity to gut microbial dysbiosis and enrichment of metabolically beneficial taxa following probiotic intervention^27^. Differences in the magnitude and pattern of microbial responses across studies may reflect variation in diet composition, obesity models, baseline microbiota structure, and analytical approaches. *Bacteroidetes*, elevated by probiotic intake, contribute to a beneficial gut environment by producing SCFA, enhancing bile acid metabolism, modulating immune responses, and anti-inflammatory signaling^28^. At the cecal species level, sample administration significantly enriched *Akkermansia muciniphila*, a species known for its metabolic benefits^29^, along with *L. lactis* subsp. *lactis*. The preferential detection of WiKim0124 in the cecum may reflect a nutrient-rich and metabolically active environment in which fermentable substrates, dietary matrix effects, and synbiotic-like interactions support strain persistence, promote local fermentation and SCFA-related metabolism^30,31^, and thereby contribute to probiotic efficacy. Notably, *Lactococcus lactis* subsp. *lactis* was scarcely detected in fecal samples, implying that its functional activity is predominantly localized to the upper colon, particularly the cecum, where it may exert beneficial metabolic effects prior to clearance from the gastrointestinal tract. Collectively, these findings support a mechanistic framework in which microbial functional modulation reshapes the gut metabolic environment, thereby enhancing host fatty acid oxidation and reducing lipid accumulation, ultimately contributing to improved obesity-related phenotypes. To elucidate microbial functional contributions, predictive functional profiling was performed using metagenomic shotgun sequencing. Enrichment of ribosomal- and transferase-associated pathways in the cecal microbiota was observed across all treatment groups. In parallel, EC profiling of fecal samples revealed elevated activities of transferases, synthases, and ligases following both WiKim0124 and SW01 administration. These findings suggest that increased microbial translational and biosynthetic activity—such as enhanced production of polyamines and SCFA^32^—may contribute to reshaping the gut metabolic environment, promoting fatty acid oxidation, reducing lipid accumulation, and ultimately supporting host lipid homeostasis^33^. In the HFD+HSuc model, UniRef pathway enrichment patterns in the cecal microbiota were comparable between the WiKim0124 and SW01 groups. However, fecal EC richness was significantly higher in the WiKim0124 group. These differences likely reflect compartment-specific microbial ecology and metabolic activity between cecal and fecal communities, whereas discrepancies between UniRef- and EC-based analyses may arise from differences in annotation depth and functional resolution, providing complementary perspectives on microbiome function. Notably, SW01 selectively enhanced Transferase and Other EC functions, while Synthase and Ligase categories showed no significant changes. SCFA-related enzyme profiling showed the lowest number of upregulated enzymes in the HFD model and the highest in the HFD + HSuc model. Distinct enzymes responded to either WiKim0124 or SW01 treatment, highlighting treatment-specific modulation of microbial metabolism. These results suggest that WiKim0124 modulates gut microbial metabolism in each dietary obesity model, regardless of formulation additives, consistent with its effects on host lipid metabolism–related protein expression. In line with these observations, SW01 showed a broadly similar anti-obesity efficacy profile to the original WiKim0124 preparation despite a ten-fold lower viable cell dose, suggesting preservation of efficacy following formulation, although the impact of dose differences on relative potency warrants further investigation. Several limitations of this study should be acknowledged. First, the sample size and targeted protein analyses may limit statistical power for some comparisons. Second, although the mouse models provide mechanistic insight, extrapolation to human obesity remains limited. Third, metagenomic functional annotations provide indirect evidence of microbial metabolic potential and were not validated by direct metabolite or enzyme activity measurements. Finally, causal studies such as microbiota transplantation or depletion approaches were beyond the scope of this work and warrant further investigation to clarify the mechanistic relationships between microbial functional changes and host metabolic improvement.

Collectively, administration of WiKim0124 cells significantly reduced body weight gain and fat accumulation in HFD-, HSuc-, and HFD+HSuc-induced obese mice, while also improving serum lipid profiles and alleviating hepatic lipid accumulation. These outcomes reflect the restoration of lipid homeostasis via regulation of key metabolic markers involved in fatty acid synthesis and oxidation. SW01—a formulated preparation containing WiKim0124—robustly activated lipid metabolism, as evidenced by increased expression of PPARα, CPT-1α, UCP2, and pHSL, alongside reduced FAS expression, particularly in fatty acid oxidation pathways. Furthermore, Shotgun metagenomic profiling of the gut microbiota based on cecal and fecal metagenomic profiles revealed a significant increase in the abundance of SCFA-related enzyme annotations following treatments. Collectively, our results suggest that WiKim0124, whether administered directly or in a formulated form (SW01), shows potential to improve obesity-related metabolic dysfunction in association with modulation of host lipid pathways and changes in gut microbiota, although the mechanistic relationships linking these effects require further verification. The beneficial effects observed across various dietary stress models highlight the potential of WiKim0124 as a functional probiotic for use in dietary supplements. Further studies are needed to clarify the specific role of formulation additives in contributing to its efficacy.

## Methods

### Bacterial strains

*L. lactis* WiKim0124 (GenBank ID: MZ424472.1), which had been previously isolated from kimchi, was utilized in this study. WiKim0124 was stored at −80 °C in De Man, Rogosa, and Sharpe (MRS) broth containing 15% (v/v) glycerol for preservation. Before conducting the experiment, the frozen cultures were revived by incubating them in MRS broth at 30 °C for 24 h. The bacterial cells were then collected by centrifugation at 6000×*g* for 10 min at 4 °C and washed twice with phosphate-buffered saline (PBS) for use in further procedures.

### Formulation of the test dose

The WiKim0124 preparation contained 1.2 × 10^11^ CFU of viable cells per gram (ShinWoo Corporation, Jinju, Korea). For the formulated product (SW01), WiKim0124 was blended with formulation additives to deliver a daily dose of 1 × 10^9^ CFU. All test materials were lyophilized to powders with identical appearance. Viable cell counts were determined by serial dilution and plate counting on MRS agar after incubation at 30 °C for 48 h. Viable counts were reconfirmed after rehydration prior to preparation of gavage doses.

### Cell culture and adipogenic differentiation

Mouse 3T3-L1 cells (ATCC CL-173) were obtained from the American Type Culture Collection and cultured at 37 °C in a 5% CO_2_ atmosphere using basal medium (BM), which consisted of high-glucose Dulbecco’s Modified Eagle Medium (DMEM; Gibco, USA) supplemented with 10% fetal bovine serum and 1% antibiotic–antimycotic solution. For adipogenic differentiation, cells were seeded at a density of 1.5 × 10^4^ cells/mL in 6-well culture plates. Cell numbers were determined using a hemocytometer with trypan blue exclusion prior to seeding, and seeding densities were adjusted accordingly. Cells were treated with DMEM containing the 3T3-L1 Differentiation Kit (Sigma-Aldrich, St. Louis, MO, USA), following the manufacturer’s instructions. Briefly, 3T3-L1 preadipocytes were cultured to confluence, followed by 48 h incubation before differentiation induction. Cells were then incubated in differentiation medium for 3 days, followed by maintenance medium replaced every 2–3 days until lipid accumulation was observed. Samples were added with the differentiation medium and replenished at each medium change. Bacterial cells were harvested, resuspended in PBS, sonicated, and centrifuged as described^13^. The resulting supernatants were filter-sterilized using a 0.45 µm filter, lyophilized, and used in anti-adipogenesis assays. WiKim0124 or SW01 lysate was added to the culture medium at concentrations ranging from 3.91 to 31.25 mg/mL. HepG2 cells (ATCC HB-8065) were also obtained from the American Type Culture Collection and maintained in DMEM supplemented with 10% fetal bovine serum and 1% antibiotic–antimycotic (Gibco, USA) at 37 °C in a 5% CO₂ humidified incubator. Free fatty acids (FFAs; oleic acid and palmitic acid in a 2:1 ratio) were dissolved in isopropyl alcohol. Once HepG2 cells reached 80% confluency, they were cultured in serum-free DMEM containing 1% bovine serum albumin (BSA). WiKim0124 lysate was added at concentrations of 1, 2, 3, and 4 mg/mL to FFA-induced cells (1 mM) and incubated for 24 h. Control cells received treatment with 1% BSA alone.

### Oil red O staining

For cytotoxicity assessment, 1 × 10^4^ cells/well were seeded in 96-well plates and exposed for 48 h to WiKim0124 lysates (1.25–20 mg/mL) or SW01 samples (3.91–62.5 mg/mL). SW01 concentrations were prepared by two-fold serial dilution from a 125 mg/mL stock solution and are expressed as mg lyophilized sample equivalent per mL of culture medium. Cell viability was measured using an XTT [2,3-bis(2-methoxy-4-nitro-5-sulfophenyl)-2H-tetrazolium-5-carboxanilide disodium salt] assay, as previously described^13^. Adipogenic differentiation was assessed using Oil Red O staining, following a previously described protocol^34^. In brief, cells were fixed in 10% formaldehyde prepared in phosphate-buffered saline (PBS), rinsed with PBS, and then stained with Oil Red O solution (Sigma-Aldrich, St. Louis, MO, USA) for 10 min. After staining, cells were repeatedly washed with distilled water. To quantify lipid accumulation, the stained lipid droplets were extracted using 100% isopropanol for 15 min, and the optical density (OD) of the resulting solution was measured at 517 nm.

### Western blot assays

Proteins were extracted from cells and tissues using RIPA buffer (Thermo Fisher Scientific, Monza, Italy) supplemented with phosphatase and protease inhibitors. After quantification, 10–60 μg of total protein from each sample was separated by 10% sodium dodecyl sulfate polyacrylamide gel electrophoresis (SDS-PAGE). The resolved protein bands were then transferred onto PVDF membranes, which were subsequently blocked with 5% skim milk for 1 h at room temperature. After washing, the membranes were incubated overnight at 4 °C with primary antibodies targeting carnitine palmitoyltransferase 1α (CPT-1α), peroxisome proliferator-activated receptor γ (PPARγ), peroxisome proliferator-activated receptor α (PPARα), AMP-activated protein kinase (AMPK), fatty acid synthase (FAS), acetyl-CoA carboxylase (ACC), and phosphorylated hormone-sensitive lipase (pHSL). Following three additional washes, membranes were incubated with horseradish peroxidase (HRP)-conjugated anti-mouse or anti-rabbit secondary antibodies in 5% skim milk for 1 hour at room temperature. Immunoreactive bands were visualized using an ECL Western blot detection kit (Thermo Fisher Scientific, Monza, Italy) and imaged with a ChemiDoc system (Bio-Rad, Hercules, CA, USA). The results were normalized to β-actin or GAPDH as a loading control.

### Animals

All animal experiments were conducted in accordance with the Guide for the Care and Use of Laboratory Animals by the National Academy of Sciences and the National Institutes of Health. The animal care procedures and experimental protocols were reviewed and approved by the Institutional Animal Care and Use Committee of the World Institute of Kimchi (WIKIM IACUC No. 202339). Six-week-old male C57BL/6 J mice were obtained from Orient Bio (Seongnam, Korea) and housed in ventilated cages under controlled temperature and humidity conditions (four mice per cage), with free access to food and either distilled water or 15% sucrose solution. After a 7-day acclimation period, the mice were randomly divided into eight groups (*n* = 8 per group): (i) Normal control (NOR): fed a standard diet (Research Diet Inc., D10001; 10 kcal% fat) and distilled water, (ii) High-fat diet (HFD): fed a high-fat diet (Research Diet Inc., D12492; 60 kcal% fat) and distilled water, (iii) HFD + WiKim0124: HFD-fed mice treated with *L. lactis* WiKim0124, (iv) High-sucrose diet (HSuc): standard diet with 15% sucrose in drinking water, (v) HSuc + WiKim0124: HSuc-fed mice treated with *L. lactis* WiKim0124, (vi) HFD + HSuc: combination of HFD and 15% sucrose in drinking water, (vii) HFD + HSuc + WiKim0124: HFD and sucrose-fed mice treated with *L. lactis* WiKim0124, (viii) HFD + HSuc + SW01: HFD and sucrose-fed mice treated with the formulated *L. lactis* WiKim0124 (SW01). SW01 was only evaluated in the HFD + HSuc. An overview of the experimental schedule is presented in Fig. 2A. Feed intake and body weight were recorded weekly. All test materials were administered orally once daily at a dose of 10 mL/kg body weight for 6 weeks. At the end of the study, mice were anesthetized using CO_2_, and blood and tissue samples were collected and stored at −80 °C for further analysis.

### Dietary dosage information

*L. lactis* WiKim0124 live cells were lyophilized following mass cultivation. The WiKim0124 powder was rehydrated in sterile distilled water for 1 hour at room temperature to achieve a final concentration of 1 × 10^10^ CFU 200 µL^−1^ d^−1^ for oral gavage. In the SW01 treatment group (HFD + HSuc + SW01), mice received a daily oral dose of 10 mg/kg body weight, equivalent to 1 × 10^9^ CFU of WiKim0124. The SW01 formulation contained approximately 1 × 10^11^ CFU/g viable cells, comparable to the original WiKim0124 raw material (1.2 × 10^11^ CFU/g).

### Histological analysis

Liver and epididymal fat tissues were collected and fixed in 10% neutral buffered formalin for histological examination, following the protocol described in a previous study^13^. After fixation, the tissues were embedded, sectioned, and stained with hematoxylin and eosin (H&E). Stained tissue sections were observed under an optical microscope (Olympus, Japan) at 20× magnification to evaluate morphological changes.

### Body composition

Body composition analysis was conducted at weeks 0, 6, and 12 using the EchoMRI system (Echo Medical Systems, Houston, TX, USA), which quantitatively measures total body fat and lean mass. To obtain detailed body composition images, dual-energy X-ray absorptiometry (DXA) scanning was performed on all mice using a system from OsteoSys (Seoul, Korea). Mice were anesthetized and positioned prone on the scanning platform with limbs and tail fully extended. In the resulting color-coded images, adipose tissue and lean tissue appeared as red and green, respectively. Image acquisition and data analysis were performed using Insight software (Version 1.0.6; OsteoSys).

### Plasma biochemical indicators

Plasma levels of triglycerides (TG), total cholesterol (T-CHO), high-density lipoprotein cholesterol (HDL-C), low-density lipoprotein cholesterol (LDL-C), aspartate aminotransferase (AST), and alanine aminotransferase (ALT) were measured using the FUJI DRI-CHEM 7000 analyzer (Fujifilm, Tokyo, Japan), following the manufacturer’s instructions.

### Library preparation and sequencing

Fecal and cecal samples (0.1 g each) were collected at week 12 to analyze microbial shifts associated with dietary intervention and WiKim0124 administration. All samples were immediately snap-frozen on dry ice and stored at −80 °C until further processing. For DNA extraction, samples were homogenized using a bead-beating grinder and lysis system (Cycle 2, FastPrep-24; MPBIO, Seoul, Korea). Genomic DNA was extracted using either the SPINeasy DNA Pro Kit for Soil (MP Biomedicals) or the Maxwell RSC PureFood GMO and Authentication Kit (Promega), following the manufacturers’ instructions. DNA concentration was quantified using a Qubit 2.0 fluorometer (Invitrogen). Sequencing libraries were prepared with the NEBNext Ultra II FS DNA Library Prep Kit for Illumina (NEB) according to the protocol designed for inputs ≤100 ng. Metagenomic sequencing was performed on the NovaSeq 6000 platform (Illumina) using 2 × 150 bp paired-end reads with the 300-cycle NovaSeq 6000 Reagent Kit.

### Taxonomic profiling

Taxonomic profiling was performed using Kraken2^35^ with a pre-built core gene database consisting of k-mers (k = 35) derived from reference genomes in the EzBioCloud database (www.ezbiocloud.net). Initially, potential bacterial and archaeal species were identified for each raw metagenomic sample. A custom Bowtie2^36^ database was then constructed using the core genes of the identified candidate species. Raw reads were aligned to this database with the parameter and a quality threshold of Phred33. The output BAM files were processed using SAMtools^36^ for conversion and sorting, and Bedtools^37^ was used to calculate coverage. To minimize false positives, only species with at least 25% total coverage across their core genes were retained using an in-house script. Species abundance was calculated based on the number of mapped reads, and normalized abundances were derived using the total length of core genes for each species.

### Functional profiling

Functional profiling was conducted using HUMAnN3^38^ to identify microbial metabolic pathways and molecular functions. Pathway abundance was estimated, and sequencing depth was normalized using Reads Per Kilobase Million (RPKM) to ensure accurate cross-sample comparisons.

### Statistical analysis

Statistical significance was determined using Tukey’s honest significant difference (HSD) test implemented via the “*agricolae*” package in R for group comparisons. A *p*-value of <0.05 was considered statistically significant. All experiments were conducted in triplicate, and data are presented as mean ± standard deviation (SD).

## Supplementary information

Supplementary information

## Data Availability

The datasets generated and/or analyzed during the current study are not publicly available due to institutional data management policies and ongoing collaborative analyses but are available from the corresponding author on reasonable request.
