# Supplementary material for: Efficacy of Lactococcus lactis WiKim0124 in Fat-, Sucrose-, and Fat/Sucrose-Induced Obesity Models
Source: NPJ Sci Food. 2026 Jun 4;10:270. doi: 10.1038/s41538-026-00915-3 (PMC13550450; doi:10.1038/s41538-026-00915-3)
Supplement: Supplementary file 1 [file 41538_2026_915_MOESM1_ESM.pdf]

# Efficacy of *Lactococcus lactis* WiKim0124 in Fat-, Sucrose-, and Fat/Sucrose-Induced Obesity Models

Moeun Lee[1], Daun Kim[2,3], Jung Hee Song[1], Shin Jae Park[4], and Ji Yoon Chang[5,6*]

[1]Sustainable Distribution Research Group, World Institute of Kimchi, Gwangju 61755, Korea

[2]Intelligent Fermentation Research Group, World Institute of Kimchi, Gwangju 61755, Korea

[3]Department of Food and Nutrition, Chosun University, Gwangju 61452, Republic of Korea

[4]ShinWoo Co., Ltd. (Headquarters), 991 Warasan-ro, Munsan-eup, Jinju-si, Gyeongsangnam-do 52839, Republic of Korea.

[5]Division of Applied Life Science (BK21), Institute of Agriculture and Life Science, Gyeongsang National University, Jinju 52828, Republic of Korea

[6]Institute of Smart Space Agriculture (ISSA), Gyeongsang National University, Jinju, Gyeongnam 52828, South Korea

[*]**Correspondence**: jychang@gnu.ac.kr (J.Y.C.)

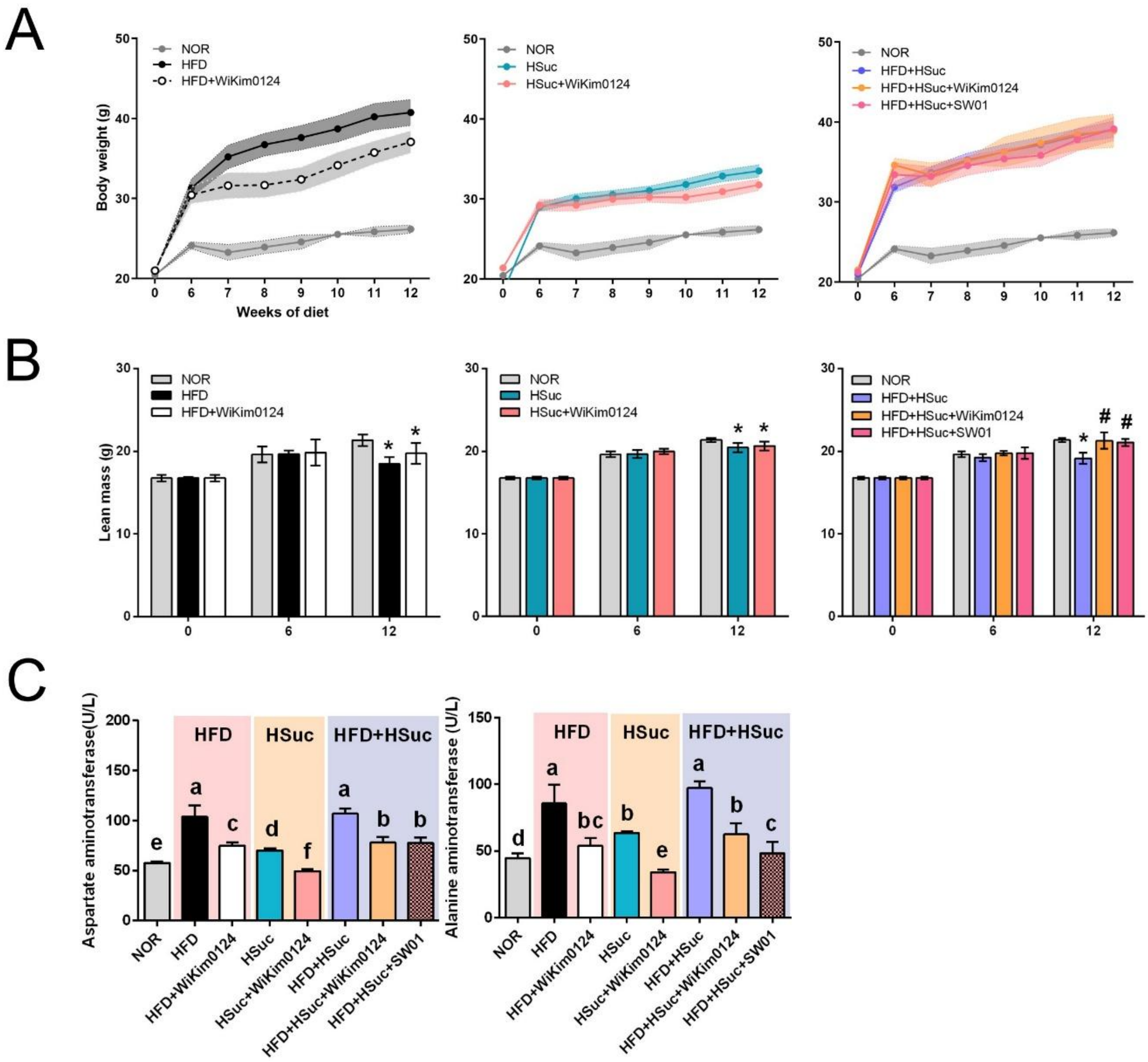


**Fig. S1. Effects of WiKim0124 and SW01 administration on physiological and biochemical parameters in HFD-, HSuc-, and HFD+HSuc-induced obese mice.** (A) Changes in body weight over the 12-week experimental period. (B) Lean mass measured at weeks 0, 6, and 12. (C) Plasma levels of aspartate aminotransferase (AST) and alanine aminotransferase (ALT). Values are presented as mean ± SD (n = 8). Different letters indicate statistically significant differences among groups ($p < 0.05$, one-way ANOVA followed by Tukey's HSD test).

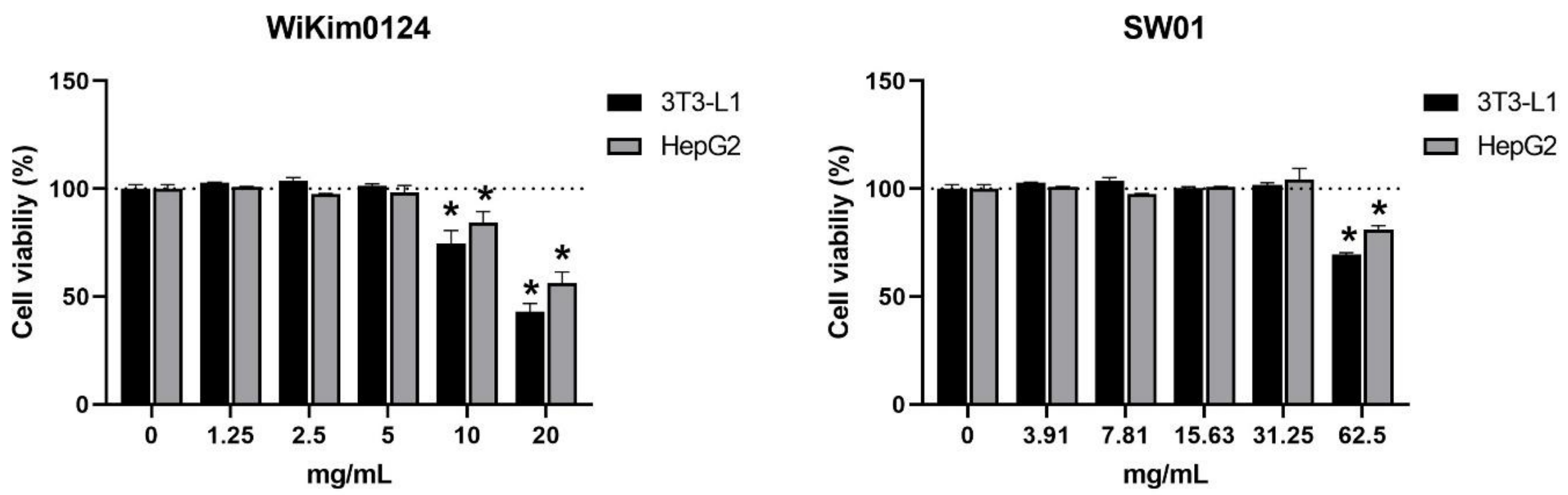


**Fig. S2. Cytotoxicity assessment of WiKim0124 and SW01 in 3T3-L1 and HepG2 cells.** Cell viability was determined by XTT assay after 48 h exposure to WiKim0124 lysates (1.25–20 mg/mL) or SW01 (3.91–62.5 mg/mL). Data are presented as mean ± SD (n = 3). *$p < 0.05$ versus untreated control.
